# Chronic Hand Eczema, Real World, and Patient Centricity: A Narrative Review

**DOI:** 10.2340/actadv.v105.42596

**Published:** 2025-04-02

**Authors:** Sonja MOLIN, Emma GUTTMAN-YASSKY, Jacob P. THYSSEN, Anthony BEWLEY

**Affiliations:** 1 Division of Dermatology, Queen’s University, Kingston, Canada; 2 Department of Dermatology, Venerology and Allergy, Charité-Universitätsmedizin Berlin, Berlin, Germany; 3 Icahn School of Medicine at Mount Sinai, New York, USA; 4 LEO Pharma A/S, Ballerup, Denmark and Department of Dermatology, Bispebjerg Hospital, University of Copenhagen, Denmark; 5 Barts Health NHS Trust & QMUL, London, UK

**Keywords:** allergic contact dermatitis, contact dermatitis, hand eczema, chronic hand eczema, occupational skin diseases, quality of life

## Abstract

Chronic hand eczema is a multifactorial, fluctuating, inflammatory skin disease of the hands and wrists, defined as hand eczema that persists for at least 3 months or recurs at least twice within 1 year. Chronic hand eczema is heterogeneous in nature with different clinical manifestations. This chronic condition can significantly impact daily activities and health-related quality of life for patients, including notable physical, psychosocial, occupational, and socioeconomic burdens. However, awareness of the disease and the patient experience remains poor. This review aims to improve understanding of chronic hand eczema in the context of its underlying pathogenesis, clinical subtypes and presentation, and risk factors. Additionally, an overview of the patient experience of the disease, including health-related quality of life and socioeconomic implications, is provided. Improved understanding of chronic hand eczema will support optimal patient care by empowering healthcare providers to more quickly and correctly diagnose this disease with high unmet treatment needs and subsequently offer appropriate holistic care.

## SIGNIFICANCE

This review aims to raise awareness and improve understanding of chronic hand eczema, an inflammatory skin disease with significant quality of life and socioeconomic impacts and high unmet treatment needs. We provide concise overviews of the current state of understanding in chronic hand eczema pathogenesis, clinical subtypes and presentation, risk factors, patient and societal disease burdens, and future perspectives on unmet needs to improve patient care. By improving the understanding of chronic hand eczema, clinicians can more quickly and correctly diagnose the disease and subsequently offer appropriate holistic clinical care.

Hand eczema (HE) is a multifactorial, heterogeneous, fluctuating, inflammatory disease of the hands and wrists characterized by itching, skin pain, erythema, oedema, oozing, crusting, papules, vesicles/bullae, and in chronic cases lichenification, hyperkeratosis, scaling, and fissures (1). In a 2021 survey of Danish adults, 13.3% of respondents had a 1-year prevalence of HE, with 35.1% of those reporting moderate to severe disease and 82.6% reporting chronic hand eczema (CHE, or HE lasting > 3 months, or recurring > 2 times within 1 year), underscoring the large burden of CHE, which may be underestimated given that not all individuals with the disease seek treatment (2, 3). An accurate measure of CHE prevalence is challenging to obtain as aetiology has driven international disease classification coding, rather than CHE as an entity (4).

Despite CHE being a common skin condition with high impact on health-related quality of life (HRQoL), including notable negative influence on the ability to work in certain occupations, awareness of the disease remains poor (5). Moreover, correctly diagnosing CHE can be challenging for healthcare providers (HCPs) because other skin diseases with similar characteristics may present on the hands/wrists, such as psoriasis, tinea, or palmoplantar pustulosis; this may lead to inadequate management and contribute to chronicity (4). Recent expert reviews calling attention to the challenges of diagnosing CHE and appropriate management/treatment options suggest a growing appreciation of the unmet needs for patients with CHE and call for improved insights and clinical evidence given the high burden of disease (3).

The aims of this review are to improve the understanding of CHE in the context of its underlying pathogenesis, clinical subtypes and presentation, and risk factors, and to illustrate the patient experience of the disease, including HRQoL implications and psychosocial comorbidities.

## DEFINING CHRONIC HAND ECZEMA

### Defining chronicity

CHE is defined as HE that persists for more than 3 months, or recurs 2 or more times within 1 year (1). There is a need for consistent, accurate, and routine use of the term “CHE” in clinical practice to further improve correct diagnosis, together with validated tools to assess disease severity and recurrence in clinical studies of this disease with high unmet treatment needs (3, 4, 6).

### Understanding complex pathogenesis, presentation, and prevention

CHE is a heterogenous condition, with a wide range of aetiologies, clinical manifestations, and subtype classification systems (7). The pathogenesis of CHE is dependent on the aetiology and subtype of the HE, with each aetiology/subtype having a unique immune signature (7). Identifying CHE is complex due to this wide variety in presentation, the phenomenon of overlapping subtypes, similarities to other skin disorders, and the disconnect between morphology and aetiology (3, 8). Further complexity derives from the multifactorial pathogenesis of CHE, which involves disruption of the skin barrier, dysregulation of inflammatory responses, alterations of the skin microbiome, and exogenous triggers, e.g., exposure to allergens or irritants (3, 9).

Recent studies show that CHE is associated with mixed Type 1/Type 2 systemic pathway activation and immune dysregulation (10, 11). Multiple pro-inflammatory cytokines associated with CHE pathogenesis signal through Janus kinase (JAK)-signal transducer and activator of transcription (STAT) pathways (12). JAK/STAT signalling plays a key role in the immune dysregulation, skin barrier disruption, and subsequent microbiome dysbiosis observed in patients with CHE (9, 13). Interferon (IFN) signalling downstream of JAK/STAT may be of particular importance, with a recent transcriptional analysis examining differentially expressed genes between vesicular CHE and atopic dermatitis (AD) showing that IFN signalling and necroptosis were significantly greater in vesicular CHE compared with AD (14).

While several classification approaches have previously been proposed, there is no universally accepted classification of CHE subtypes (3). Common aetiological subtypes relating to a particular trigger include irritant contact dermatitis, allergic contact dermatitis, and atopic HE; protein contact dermatitis/contact urticaria is another subtype that infrequently occurs and can be difficult to diagnose (1, 4, 15). Irritant contact dermatitis is characterized by skin impairment due to an irritant, e.g., through wet work, and is associated with innate immune and Th1/Th17 inflammation (7). Allergic contact dermatitis is a delayed, type IV hypersensitivity reaction, which may arise in some individuals with a contact allergy upon repeat exposure to the antigen (7). A positive patch test reaction to an allergen is a prerequisite for allergic contact dermatitis diagnosis. Common contact allergens are metals (Th1/Th17 profile), or rubber accelerators/fragrances (Th2/Th22 profile) (7, 16). Atopic HE occurs in patients with a medical history of atopic eczema with-out documented irritant exposure (Th2/Th22 profile) (7, 16). Protein contact dermatitis/contact urticaria is an immediate, type I and type IV hypersensitivity reaction caused by exposure to proteins (food, latex, or other biological material) (17). Morphological subtypes include hyperkeratotic, acute recurrent vesicular, and nummular eczema; pulpitis may also occur (1, 15). CHE with the presence of eczematous lesions on other body locations has been associated with more severe disease/poor outcomes (18, 19). The feet are a frequent site for disseminated eczematous lesions, occurring in up to 30% of patients with CHE (18). The cause of disseminated eczematous lesions in patients with CHE is unknown; however, one possibility is a process known as autoeczematisation, or the id reaction (20). In patients experiencing autoeczematisation, generalized dermatitis occurs days or weeks after a local exacerbation of a chronic inflammatory skin disease, such as CHE (20). Other possible causes may include AD, spreading allergic contact dermatitis, concomitant exposure of other body sites to allergens/irritants, or other skin diseases.

CHE classification, and therefore identification, could be improved by understanding the distinct immune signatures between subtypes, resulting in targeted treatment and counselling options. A recent study profiling systemic biomarkers suggested a potential for identifying certain CHE subtypes based on their Type 1/Type 2 inflammatory plasma signature (10); for example, in very severe, chronic allergic contact dermatitis, Type 1 (CXCR3 ligands CXCL9–11) and Type 2 inflammatory markers (CCL17 and MCP-4/CCL13) were upregulated compared with healthy controls. However, biomarker profiling to clearly differentiate subtypes is not yet available/sufficiently established for clinical practice (7). Until biomarker profiling is readily available, awareness of the aforementioned subtypes and their related environmental/occupational triggers is needed for primary prevention of CHE in at-risk individuals (i.e., behavioural change to improve skin protection) (3). Secondary prevention may include the introduction of emollients/barrier creams and avoidance of specific allergens/irritants for those with mild disease (3).

## RISK FACTORS FOR CHRONIC HAND ECZEMA

AD is well established as the strongest known risk factor for HE, especially in children (6, 21–23). The presence of AD in childhood, particularly AD that is persistent or severe, may increase the risk of developing HE (1). Other risk factors for HE include female sex, prior history of HE, low age at onset of HE, having a contact allergy, cold/dry weather, decreased indoor humidity, and occupational or domestic exposure to allergens and irritants with wet work/mixed exposures (e.g., hairdressers, cleaners, healthcare workers) (1, 6). HE occurrence among females is ~1.5–2 times higher than among males (3, 6, 24). This has generally been attributed to differences in environmental exposures, including more frequent hand washing and time spent on domestic/occupational wet work by females compared with males, rather than differences in skin physiology/susceptibility (3, 6). Intensity of wet work (defined as having wet hands or wearing occlusive gloves for ≥ 2 h per day, or hand washing ≥ 20 times per day) has been found to be directly related to HE risk (25). Environmental factors may account for up to 59% of the aetiology of HE, independent of AD (26). Genetic risk factors may account for the remaining 41% (26). Loss-of-function mutations in the gene encoding the skin structural protein filaggrin may predispose people with AD to develop CHE, characterized by early onset and persistence (1, 27). In a study of over 2,000 adults, 18.4% of the participants with both filaggrin gene mutations and AD reported a lifetime prevalence of both foot and persistent hand dermatitis (27). Once someone has HE, it should be treated quickly to reduce the risk of progression to CHE (1).

## WHAT IS IT LIKE TO LIVE WITH CHRONIC HAND ECZEMA?

### Physical and psychosocial burdens of chronic hand eczema

CHE often is a long-lasting disease, with a median duration of 11–16 years, that may “flare” in episodes/periods when symptoms are worsened, with inflammation-associated symptoms particularly heightened during the flare (6, 8). Itching and skin pain are 2 of the most common symptoms in patients with CHE and contribute to poor HRQoL (3, 8). Scratching due to itch has been cited by patients as contributing to other symptoms (e.g., bleeding, erythema, and flaking) (8). Skin pain has often resulted from signs of CHE, such as fissures (1). Moreover, many individuals with CHE also have comorbid skin diseases, such as AD (6), and may present with disseminated eczematous lesions on parts of the body other than hands (19, 20).

**Fig. 1:**
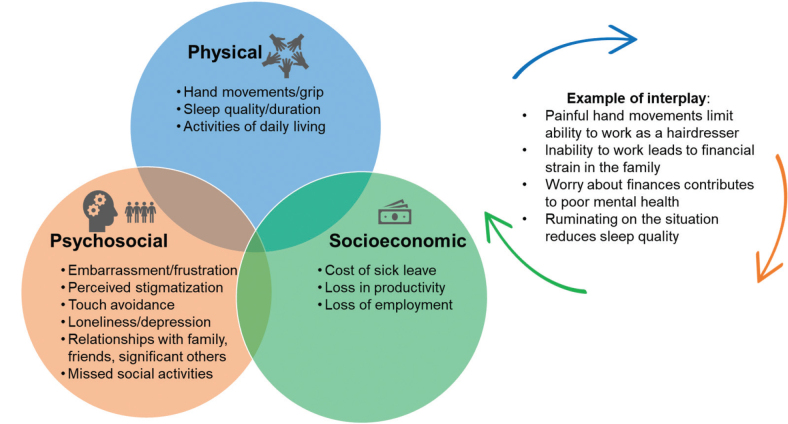
**Multiple interconnected factors contribute to psychosocial burdens/health-related quality of life for people with chronic hand eczema.** The figure includes examples based on the literature (1, 8, 28–30) and is not exhaustive of all patient experiences.

Given these disease characteristics, patients with CHE may experience challenges to their physical, psychosocial, and socioeconomic well-being, covering multiple domains of HRQoL (**Fig. 1**) (1, 2, 8). A UK survey of over 1,000 individuals with HE found that 89% were embarrassed/self-conscious about their disease, with many saying their condition affects the way they handle objects or touch people (8). In a cross-sectional, observational, multicentre study among 3,635 dermatological patients in Europe, patients with HE (*n* = 143) reported significantly greater clinical depression, anxiety disorder, and suicidal ideation compared with patients with other common skin diseases and controls (28). Psychological impacts of CHE may also be experienced by family/friends; individuals with HE have reported self-isolation and disagreements with their partner or family/friends, because they felt their disease stops them from doing certain activities (29). The various elements of psychosocial well-being may interplay with one another; for example, patients with CHE cite negative effects on mood (29), which may influence decisions concerning other aspects of life (Fig. 1).

### Socioeconomic burdens of chronic hand eczema

Given the localization of CHE on the hands, and frequent connection to particular work-related triggers, CHE can have notable impacts on employment and personal economy. A recent review of studies conducted in Europe, Australia, New Zealand, and the Americas summarized the economic burden of CHE (30). The annual societal costs per patient of CHE ranged between €1,813 (US$2,549, 30% direct costs, 70% indirect costs) and €7,738 (US$10,883, 49% direct costs, 51% indirect costs) in Europe (30).

CHE has a substantial economic impact on both the patient and society due to job loss and absenteeism (i.e., inability to attend work). Occupational skin diseases may lead to job changes and even early retirement (1); the fluctuating nature of CHE and possibility for symptoms to persist/flare up over many years puts patients at risk of long-term sick leave and, ultimately, job loss. In a recent systematic review, up to 57% of patients took sick leave (mean 7.2–35 mean days per year) and up to 25% reported leaving or changing their job due to CHE (30).

For individuals who do attend work, presenteeism (i.e., attending work but with reduced performance) is a widespread issue (30). In a survey of 500 Dutch adults with CHE, 40.8% reported presenteeism in the past year, with 22.7% working when they ought to have rested/recovered because they were “afraid of losing their job” (31). In the same study, CHE severity was significantly associated with presenteeism prevalence (31). In a survey of 507 individuals in a managed care organization in the USA, there was no significant difference in work time missed between people with or without CHE; however, people with CHE had significant impairment while working (mean % ± SD scores: 26.86 ± 31.39 vs 12.68 ± 23.10, *p* < 0.001), overall work impairment (29.33 ± 31.73 vs 6.85 ± 21.45, *p* < 0.001), and activity impairment (33.78 ± 36.07 vs 17.32 ± 26.96, *p* < 0.001) relative to people without CHE (32).

### Unmet chronic hand eczema treatment needs

Current treatment options for CHE are limited, with a particular unmet need for options that are safe and efficacious for long-term management of CHE (1, 3, 7). While many patients with CHE use topical corticosteroids (TCS), it is an inadequate option for many and not suitable long term. In a survey of patients with CHE from the Danish Skin Cohort, 84.8% (614/724) reported at least 1 adverse event from the use of TCS for their CHE, with skin atrophy being the most common adverse event (33). In a multicentre study of 819 patients with CHE, 12.5% (*n* = 102) of the overall CHE population were refractory to TCS therapy (34). Furthermore, in a survey of 724 adults with CHE in Denmark, 76% of patients indicated they would prefer a non-steroidal treatment option (33). Topical calcineurin inhibitors may also be used for CHE treatment; however, data on efficacy are limited (3, 7). In addition to topical therapy, systemic therapies have been suggested for severe/refractory cases of CHE, including alitretinoin (dependent on country approval), cyclosporine A (off-label), methotrexate (off-label), azathioprine (off-label), and acitretin (off-label) (1, 3, 7). However, real-world studies for these systemic therapies suggest poor drug survival rates, with main reasons for treatment discontinuation being adverse events or lack of efficacy, highlighting the need for additional long-term treatment options for CHE (35–38). Emerging targeted biologic and synthetic treatments, including interleukin (IL)-4/IL-13 inhibitors developed/approved for AD and topical/oral JAK inhibitors, have shown promising results for the treatment of CHE in clinical trials and have recently been expertly reviewed (3, 39). Of these, the topical pan-JAK inhibitor delgocitinib cream 20 mg/g was approved by the European Medicines Agency in September 2024 for the treatment of moderate to severe CHE in adults for whom TCS are inadequate or inappropriate (40). More studies to better understand existing options and to develop new therapeutics would be beneficial for offering more choices for people with CHE.

## DISCUSSION

CHE is a common skin disease with significant HRQoL and socioeconomic impacts; however, complexity in accurately identifying CHE contributes to treatment gaps. Improved understanding of the disease would empower HCPs to more quickly identify individuals at risk of CHE or already having CHE (as opposed to another skin disorder) and subsequently offer appropriate prevention and/or treatment.

While CHE and AD have some common presentations, and may be associated with each other, they are distinct diseases with different treatment approaches and transcriptional differences (1, 4, 14). Understanding CHE as a disease in its own right, requiring specific treatments, will support optimal patient care (4).

To begin treatment, the European Society of Contact Dermatitis (ESCD) 2022 guidelines recommend taking a medical history and performing a clinical examination to obtain a proper diagnosis and exclude other clinical manifestations that are similar to CHE, such as infection (1). Discussion with the patient should include questions to identify potential triggers and to evaluate the patient’s psychological well-being, physical functioning, and impact on work. A patch test should be pursued where possible for all patients who have had HE for more than 3 months, or who are non-responsive to appropriate treatment, or if there is concern regarding contact allergy (1). This well-rounded approach would aid in both mitigating triggers and ensuring patients are supported in all domains of their life as best as possible. Holistic care should also include a discussion of appropriate treatment options, which hopefully will be expanded and improved upon as emerging options become available (39). In the future, improved biomarker assessments within clinical practice and more refined understanding of the molecular underpinnings of disease could aid with identification of CHE subtypes or distinguishing from other skin diseases.

## Data Availability

Data sharing not applicable to this article as no datasets were generated or analysed during the current review.
